# Cell-Laden 3D Printed GelMA/HAp and THA Hydrogel Bioinks: Development of Osteochondral Tissue-like Bioinks

**DOI:** 10.3390/ma16227214

**Published:** 2023-11-17

**Authors:** Shahrbanoo Jahangir, Jana Vecstaudza, Adriana Augurio, Elena Canciani, Liga Stipniece, Janis Locs, Mauro Alini, Tiziano Serra

**Affiliations:** 1 AO Research Institute Davos, 7270 Davos, Switzerland; shahrbanoo.jahangir@aofoundation.org (S.J.); adriana.augurio@aofoundation.org (A.A.); mauro.alini@aofoundation.org (M.A.); 2 Rudolfs Cimdins Riga Biomaterials Innovations and Development Centre of RTU, Institute of General Chemical Engineering, Faculty of Materials Science and Applied Chemistry, Riga Technical University, Pulka 3, LV-1007 Riga, Latvia; jana.vecstaudza@rtu.lv (J.V.); liga.stipniece@rtu.lv (L.S.); 3 Baltic Biomaterials Centre of Excellence Headquarters, LV-1007 Riga, Latvia; 4 Department of Health Sciences, Center for Translational Research on Allergic and Autoimmune Diseases (CAAD), University of Piemonte Orientale UPO, Corso Trieste 15/A, 28100 Novara, Italy; elena.canciani@uniupo.it

**Keywords:** cell-laden hydrogel, 3D printing, GelMA, tyramine, hyaluronic acid, chondrocyte, osteoblasts, cell viability, osteochondral regeneration

## Abstract

Osteochondral (OC) disorders such as osteoarthritis (OA) damage joint cartilage and subchondral bone tissue. To understand the disease, facilitate drug screening, and advance therapeutic development, in vitro models of OC tissue are essential. This study aims to create a bioprinted OC miniature construct that replicates the cartilage and bone compartments. For this purpose, two hydrogels were selected: one composed of gelatin methacrylate (GelMA) blended with nanosized hydroxyapatite (nHAp) and the other consisting of tyramine-modified hyaluronic acid (THA) to mimic bone and cartilage tissue, respectively. We characterized these hydrogels using rheological testing and assessed their cytotoxicity with live-dead assays. Subsequently, human osteoblasts (hOBs) were encapsulated in GelMA-nHAp, while micropellet chondrocytes were incorporated into THA hydrogels for bioprinting the osteochondral construct. After one week of culture, successful OC tissue generation was confirmed through RT-PCR and histology. Notably, GelMA/nHAp hydrogels exhibited a significantly higher storage modulus (G′) compared to GelMA alone. Rheological temperature sweeps and printing tests determined an optimal printing temperature of 20 °C, which remained unaffected by the addition of nHAp. Cell encapsulation did not alter the storage modulus, as demonstrated by amplitude sweep tests, in either GelMA/nHAp or THA hydrogels. Cell viability assays using Ca-AM and EthD-1 staining revealed high cell viability in both GelMA/nHAp and THA hydrogels. Furthermore, RT-PCR and histological analysis confirmed the maintenance of osteogenic and chondrogenic properties in GelMA/nHAp and THA hydrogels, respectively. In conclusion, we have developed GelMA-nHAp and THA hydrogels to simulate bone and cartilage components, optimized 3D printing parameters, and ensured cell viability for bioprinting OC constructs.

## 1. Introduction

Cartilage is a highly specialized tissue lacking blood supply, nerve tissue, and lymphatics with a limited spontaneous regeneration capacity because of its avascular nature [1,2]. The portion located beyond the deepest part of natural cartilage tissue is known as calcified cartilage, which connects the cartilage to the underlying subchondral bone [1]. The formation of osteochondral (OC) defects, affecting superficial articular cartilage, intermediate calcified cartilage, and deep subchondral bone, is a common worldwide health problem, which can be caused by trauma, osteoarthritis, a tumor, and aging [2,3,4]. Even if various clinical treatments for OC defect repair have been performed, such as autologous chondrocyte implantation, microfracture, and mosaicplasty, such conventional approaches generally show limitations in terms of long-term correction of cartilage disorders [1]. Thus, alternative strategies for the treatment of OC defects need to be explored. Among them, the development of synthetic scaffolds through tissue engineering methodologies can be a promising solution for cartilage regeneration and repair [2].

Three-dimensional (3D) printing technology has recently gained great attention in tissue engineering (TE) thanks to its capacity to tune bulk geometry and internal structure of the desired scaffolds [3,5]. This process allows the printability of hydrogels as biomaterial ink using a layer-by-layer approach and computer-assisted design for the fabrication of solid 3D structures such as artificial implants or complex tissue [6,7]. Furthermore, it allows the fabrication of 3D tissue-engineered structures containing living cells [8,9]. Several 3D bioprinting technologies have been implemented for the development of various biological constructs, including blood vessels, liver, bone, and heart [10]. Bioinks, which are the main conduit of 3D bioprinting, are composed of one or more hydrogel materials, additives, and cells. Bioprinted cells can significantly improve the correct functioning of the fabricated 3D structures. In particular, the cell type and the number are crucial factors in bioprinting [3]. For example, it has been reported that bioprinting technology could not only improve the stiffness of the hydrogel but also promote cell adhesion, osteogenic differentiation, and bone matrix deposition using MSCs [11]. Consequently, osteoblast and chondrocyte-laden hydrogel-based 3D bioprinting is an excellent system to offer promising therapeutic strategies for bone and cartilage repair [3].

Hydrogels are commonly used as biomaterial inks in 3D bioprinting due to their mechanically supportive microenvironment, tunable physical and/or chemical properties, as well as well-documented biocompatibility, ensuring their application in tissue engineering [12,13]. In addition, owing to their porous network, hydrogels can mimic the extracellular matrix (ECM), offering an ideal microenvironment for cell migration and proliferation [14,15]. Hydrogels can be composed of natural polymers, including hyaluronic acid (HA), gelatin, alginate, collagen, and fibrin, which are suitable candidates for the development of various biomaterials-based formulations for 3D printing in TE [2]. Particularly, HA-based hydrogels have been explored for applications in 3D bioprinting [16,17]. HA is a non-sulfated glycosaminoglycan and a main component of the ECM and it is present in the connective tissues of most mammals, where it interacts with several cell surface receptors [18]. However, native HA solutions generally flow after extrusion and thus cross-linking strategies, such as enzymatic cross-linking and photo-crosslinking, need to be investigated to obtain shape retention [19]. Additionally, considering the limited adhesion capacity of the cells on HA hydrogels, the incorporation of tripeptide arginyl-glycyl-aspartic acid (RGD) is also required [20]. Among various cross-linkable HA ink formulations, a tyramine-modified hyaluronic acid (HA-Tyr) was recently introduced [21]. HA-Tyr involves a dual gelation mechanism: a standard enzymatic pre-crosslinking based on horseradish peroxidase (HRP) and hydrogen peroxide (H_2_O_2_) to a secondary cross-linking triggered by visible green light using Eosin Y (EO) as photo-initiator. The cross-linking based on HRP/H_2_O_2_ is a well-documented non-cytotoxic mechanism adopted for the gelation of different natural polymers. Likewise, EO dye has been already employed for the photo-cross-linking of various polymers [22].

Flégeau et al. demonstrated that chondrocyte-laden HA-Tyr microgels support the maturation of 3D printed cartilage-like tissue, making them promising bioinks for 3D printing application [23]. Similarly, gelatin methacrylate (GelMA) derivative has been widely used as hydrogel ink. GelMA is a major ECM component obtained from collagen hydrolysis, containing matrix metalloproteinase sequences and arginine-glycine aspartic (RGD) acids for stimulating cell adhesion and proliferation [2]. Generally, GelMA with a 50% or higher degree of functionalization is dissolved at concentrations above 5% *w*/*v* into a solution containing a photoinitiator. After the viscous ink is extruded, the printed structure is exposed to UV light and undergoes photo-crosslinking, which ensures the stability of the 3D printed hydrogel [24].

Besides the various advantages of hydrogels, the mechanical properties and low sustainability of hydrogels after 3D printing can significantly affect the retention of 3D structures for experiments in vitro and in vivo. It has been reported that the addition of fillers into the hydrogel matrix can considerably improve the mechanical properties of the 3D printed polymeric structure [5]. Specifically, hydroxyapatite (HAp) is an excellent additive to hydrogels for bone tissue engineering. Indeed, it is well known that a high content (>65% *w*/*w*) of HAp is found in the calcified cartilage or the subchondral bone [25]. HAp itself can facilitate osteoblast adhesion, proliferation, and ECM deposition [1]. According to previous studies, the inclusion of HAp into hydrogels such as GelMA can improve mechanical stability as well as decrease the degradation rate of the hydrogel [26,27]. For instance, Allen et al. showed that the addition of HAp to GelMA-gelatin hydrogel osteoblast-laden significantly decreases the hydrogel swelling, improves the capacity of the hydrogel to resist enzymatic degradation, and enhances the osteoblastic differentiation, while maintaining good cell viability [28].

In this work, we develop and study a dual bioink comprising chondrocyte-laden HA-Tyr hydrogel and osteoblast-laden GelMA/HAp hydrogel for the 3D printing of osteochondral scaffolds. We optimized the viscoelastic and printability properties of cell-laden HA-Tyr and GelMA formulations to obtain a 3D bioprinted structure with high resolution and shape fidelity. Importantly, we further demonstrate that the cell viability as well as the expression of bone and cartilage-specific genes are preserved in the 3D printed GelMA/HAp and THA constructs. These findings indicate that 3D-bioprinted cell-laden HA-Tyr and GelMA/HAp scaffolds showed not only adequate mechanical strength but also maintained a suitable 3D microenvironment for differentiation and proliferation of chondrocytes and osteoblasts, suggesting that this developed 3D-printed scaffold may have great potential application for bone and cartilage tissue repair and regeneration in vivo.

## 2. Materials and Methods

### 2.1. Synthesis and Characterization of GelMA

For functionalization of gelatin with methacryloyl groups, or synthesis of GelMA, a clear solution of 10 g of gelatin (type A, gel strength 175 g Bloom, Sigma Aldrich, St. Louis, MO, USA) was dissolved in 100 mL of phosphate buffer saline (PBS) and stirred in an oil bath at 60 °C for 30 min. Then, methacrylic anhydride (MA) was slowly added dropwise while stirring the dissolved gelatin with a magnetic stirrer. After that, the solution was stirred at 50 °C for 3 h to allow complete reaction of MA with free amine groups and then the reaction was quenched via the addition of 400 mL of warm (40 °C) PBS. Then, the mixture was dialyzed using regenerated cellulose dialysis tubing with a cutoff of 12–14 kDa against demineralized water for seven days at 40 °C. Dialyzed GelMA was frozen at −20 °C overnight, lyophilized at −80 °C, and stored at −20 °C until further use.

The degree of substitution (DOS) of the free amine groups Is determined by the final concentration of MA. DOS of GelMA with methacryloyl groups was determined using proton nuclear magnetic resonance (H-NMR) spectroscopy. To prepare the samples for H-NMR GelMA and gelatin (30 mg each) were dissolved in 1 mL of deuterium oxide (D_2_O, MW: 20.03 g/mol, Sigma-Aldrich). H-NMR spectra were collected using an NMR spectrometer from Bruker Biospin (Fällanden, Switzerland) Advance IIIHD equipped with a 5 mm BBFO probe head and a 7.0 T magnet corresponding to a 1H Larmor Frequency of 300 MHz with an automated sample changer and IconNMR (version 4.7.3) and Topspin (version 3.2) software. The DOS of GelMA was calculated using software mNova 9 (Mestrelab, Coruna, Spain). The peak of aromatic amino acids around 7.3–7.4 ppm was integrated and set to 1 as the reference value. The peak of the lysine amino groups was evaluated to determine the DOS by MA through the integration of its peak at around 2.8–3 ppm. This peak becomes smaller when the lysine amino groups are functionalized with MA and therefore, it can be compared with the peak around 2.8–3 ppm of pure gelatin.

### 2.2. Synthesis and Characterization of THA

The synthesis procedure and characterization of tyramine-modified hyaluronic acid (THA) was adapted from Petta et al., 2018 [29]. Briefly, first, 10 g of hyaluronic acid (HA, information about the chemical) were dissolved in 1 L of demineralized water under constant stirring overnight at room temperature (RT).

The HA solution was heated up to 37 °C, and 6.92 g of 4-(4,6-dimethoxy-1,3,5-triazin-2-yl)-4-methyl-morpholinium chloride (DMTMM) was added. Then, 4.34 g of tyramine hydrochloride was dissolved in 25 mL distilled water and then slowly added to the HA solution while stirring mildly for the next 24 h. For precipitation, a saturated NaCl solution (16% (*v*/*v*), in a total of 160 mL) was added dropwise, and the solution was stirred for another 30 min at RT. Then, 3 L of 96% ethanol was added dropwise, followed by 30 min of stirring. Subsequently, the solution was vacuum filtered and washed several times with 80% ethanol to dissolve the remaining NaCl residuals. Chloride assay using silver nitrate was used to confirm the absence of chlorides. At the point when no chloride was detected anymore, the precipitate was washed with 96% ethanol, followed by washing it with 99.98% ethanol. After every change in ethanol percentage, the precipitate was vacuum filtered for 20 s. Subsequently, the precipitated THA was dried under vacuum for 48 h. Finally, the final product was stored in 50 mL falcon tubes.

#### THA Characterization: H-NMR and UV–Vis Measurement

The degree of functionalization (DoF) of THA was determined using H-NMR and UV–Vis spectrometry. H-NMR spectra were performed on 30 mg of THA that were dissolved in D_2_O and later analyzed using an NMR spectrometer from Bruker Biospin (Fällanden, Switzerland) Avance III HD equipped with a 5 mm BBFO probe head and a 7.0 T magnet corresponding to a 1H Larmor Frequency of 300 MHz. Samples were measured using an automated sample changer with IconNMR (version 4.7.3) and Topspin (version 3.2) software. The software MNova 9 from Mestrelab was used to determine the DoF of THA. The integral of the peak from 1.75 to 2.1 ppm was set to three, as it contributes to three protons. The average integrals of peaks from 6.75 to 6.8 ppm and from 7.75 to 7.8 ppm was the DoF with tyramine. 

The second method to determine the DoF of THA by tyramine is based on a UV absorbance measurement. Briefly, THA was dissolved in 1 mg/mL in H_2_O and the standards were prepared with tyramine hydrochloride in H_2_O: 1000, 500, 250, 125, 25, 10, 1, 0 μg/mL. Subsequently, 100 µL per well of sample and standards were dispensed in triplicates into a UV-transparent 96-well plate then the absorbance was measured at 275 nm using a plate reader, and DoF was calculated according to the standard curve as below: 

Tyramine molar concentration c (mol/L) of the sample as:

C = (“absorbance” − “intercept”)/(“slope × 173.64 × “ 10^3^)

Note that 173.64 g/mol is the molecular weight of tyramine hydrochloride.

DoF (%) of the sample:

DoF = 100% × (C × MW (0%))/(ρ − c × (MW (100%) − MW (0%)))

MW (0%): Molecular weight of HA sodium salt (401.30 g/mol), MW (100%): Molecular weight of fully substituted THA (498.44 g/mol), ρ: Mass concentration of the dissolved sample (1 mg/mL) or simplified as: DoF = (401.30 × c)/(1 − 97.14 × c).

### 2.3. Synthesis and Characterization of nHAp

Nano-hydroxyapatite (nHAp) was synthesized by wet chemical precipitation technology from calcium oxide (CaO) and orthophosphoric acid (H_3_PO_4_) according to Reactions (1) and (2):

CaO + H_2_O → Ca(OH)_2_ (1)

10Ca(OH)_2_ + 6H_3_PO_4_ → Ca_10_(PO_4_)_6_(OH)_2_ + 18H_2_O (2)

At first, Ca(OH)_2_ (95.0–100.5%, Jost Chemicals Co., St. Louis, MO, USA) was calcined at 1100 °C for 1 h with a heating rate of 5 °C/min to obtain CaO. The obtained CaO (16.8 g) was dispersed in 2 L of deionized H_2_O (conductivity of 5.5 μS/m) and stirred at room temperature (22 ± 2 °C) for 1 h at 100 rpm in the laboratory reactor equipped with an anchor mixer (Power Control-Visc P7, IKA Eurostar, Staufen im Breisgau, Germany). Then, the synthesis mixture was heated to 45 °C and addition of 2 M H_3_PO_4_ (75%, Latvijas ķīmija Ltd., Riga, Latvia) was started with a rate of 1 mL/min using a dosing system (TITRONIC^®^ universal, Schott, Apeldoorn, The Netherlands). When the pH of the synthesis reached 7.08, the synthesis mixture was stirred for 1 h at 100 rpm. After 1 h, the obtained precipitates were left to mature overnight for 20 h. Further, the precipitates were re-stirred at 100 rpm for 30 min. Excess water was removed using vacuum filtration. Further, the precipitated nHAp was dried using spray-drying to obtain nHAp in the form of spray-dried spheres for easier powder handling. For this purpose, a tabletop spray dryer (Mini Spray Dryer B-290, Büchi, Flawil, Switzerland) was used. Prior spray-drying the wet precipitates of nHAp (moisture content 88 ± 1 wt.%) were diluted with deionized water to obtain a concentration of 0.02 mol/L. The diluted nHAp slurry was spray-dried using conditions summarized in Table 1.

The spray-dried nHAp powder was characterized before (ICP-MS, XRD, FT-IR, BET, SEM and TEM) and after (XRD and FT-IR) sintering at 1100 °C for 1 h in a muffle furnace (heating/cooling rate of 5 °C/min). Sintering at 1100 °C temperature was carried out to determine the Ca/P molar ratio of the synthesized calcium phosphate and thus its phase purity. 

An ICP-MS instrument Agilent 7700x (Agilent Technologies, Santa Clara, CA, USA) with MassHunter software (version B.01.03, Tokyo, Japan) was used for the analysis of chemical elements of the spray-dried nHAp. Samples were dissolved in a high-purity nitric acid solution (65%, ChemLab, Zedelgem, Belgium) that was diluted with deionized water (Millipore Synergy 185, Molsheim, France). Dissolution of the samples took place at RT for at least 20 min. Next, the sample containing vessels were capped and transferred to a Mars 6 microwave oven (CEM Corporation, Matthews, NC, USA) for digestion. The temperature of the microwave was raised to 150 °C within 30 min and was held at 150 °C for 30 min then the vessels were cooled. After the cooling, the samples were filtered using a 12–15 μm pore size filter (Filtres Fioroni, Ingré, France) and quantitatively transferred to volumetric flasks and diluted with deionized water.

By means of an X-ray diffraction (XRD) analysis, phase composition over a 2θ range of 10–70° was determined. This was done with an X’Pert Pro (PANalytical, Almelo, The Netherlands) diffractometer using Cu Kα (λ = 1.5406 Å) radiation produced at 40 kV and 30 mA with a step size of 0.06°, and a counting time of 200.025 s. Prior XRD analysis samples were finely ground with an agate mortar and pestle. The ground sample was put on a front-loading sample holder and gently flattened. During the XRD measurements, the sample holder was rotated. For crystalline phase identification (reference card #01-072-1243 for hydroxyapatite), the International Centre for Diffraction Data (ICDD) database PDF-2 and software HighScore (PANalytical, Almelo, The Netherlands, v4.9) was used.

A Fourier transform infrared spectrometer (FT-IR, Varian 800, Scimitar Series, Palo, Alto, CA, USA) was used for FT-IR spectra collection of finely ground samples The FT-IR spectra were recorded in the range of 400–4000 cm^−1^ with a spectral resolution of 4 cm^−1^ and by averaging 50 scans for each measurement using attenuated total reflectance (ATR) mode with an GladiATR^TM^ (Pike technologies, Madison, WI, USA) accessory. The background spectrum was deducted from each sample’s spectrum.

Brunauer–Emmett–Teller (BET) method was used to determine the specific surface area (SSA) of the spray-dried nHAp by N_2_ sorption (QuadraSorb SI Kr, Quantachrome Instruments, Boynton Beach, FL, USA). The spray-dried nHAp powders were degassed at 25 °C for 24 h right before the analysis using Autosorb Degasser AD-9 (Quantachrome Instruments, USA). Particle size d_BET_ was calculated according to formula: d_BET_ = 6/(ρ × SSA), where ρ is density and SSA is specific surface area of the material. 

The morphology of the spray-dried nHAp was studied by the field emission scanning electron microscopy (FE-SEM, Mira/LMU, Tescan, Brno, Czech Republic) at an acceleration voltage of 5 kV. First, the spray-dried nHAp was sifted onto double sided electroconductive tape attached to a standard aluminum sample holder. Excess of the sample was removed by compressed air. Then, the samples were sputter-coated in an argon atmosphere with a thin layer of gold (thickness around 15 nm) to increase the conductivity of the sample by using sputter coater K550X (Quorum technologies, UK). The size of the spray-dried spheres was measured using ImageJ Fiji (version 1.53q) software. Furthermore, for high resolution and high magnification studies on nHAp nanoparticles, a transmission electron microscope (TEM) (Tecnai GF-20, FEI, Hillsboro, OR, USA) was used at 200 kV. For TEM measurements nHAp was dispersed in ethanol and transferred onto a carbon-coated grid. The size of the nHAp particles was measured using the ImageJ software (v1.53k).

### 2.4. Preparation and Characterization of the Bioinks

#### 2.4.1. GelMA/nHAp Bioinks

GelMA/nHAp hydrogels were prepared by mixture of 1% *w*/*v* nHAp with a 10% *w*/*v* GelMA. A well-established crosslinking technique of GelMA is the photo-crosslinking with Irgacure 2959 as the photoinitiator, which triggers polymerization. First, the Irgacure 2959 powder was dissolved in PBS at 70 °C for approximately 60 min (the final concentration was 0.3% (*w*/*v*)). Then, the desired amount of GelMA was dissolved in the prepared Irgacure 2959 solution and mixed at 37 °C. Subsequently, the nHAp solution in PBS was admixed to the GelMA to obtain the ink for 3D printing. 

#### 2.4.2. THA Bioinks

The synthesized THA was dissolved in PBS solution containing 0.1 U/mL horseradish peroxidase (HRP) at final concentration of 3.5% *w*/*v* overnight at 4 °C temperature under mild rotation. After complete dissolution of THA, eosin Y (EO) was reconstituted in dimethyl sulfoxide (DMSO) and mixed with the THA precursor to a final concentration of 0.02% *w*/*v*. The enzymatic crosslinking was initiated by adding H_2_O_2_ having concentrations from 55 to 130 µM. This range of H_2_O_2_ concentrations was selected based on rheology data and printability of THA. Then, the required light crosslinking was triggered by exposing the enzymatically crosslinked THA bioink for 5 min to green light (λ = 505 nm: light source intensity = 80 mW/cm^2^).

#### 2.4.3. Rheological Characterization of the Bioinks

To investigate the viscoelastic properties of the GelMA-based hydrogel and THA hydrogel, rheological characterization was performed using amplitude sweep, frequency sweep, temperature sweep, and time sweep tests. Tests were done using MCR-302 rheometer (Anton Paar, Graz, Austria) equipped with a Peltier temperature control unit.

##### Rheological Measurements of GelMA-Based Hydrogels

The oscillatory strain sweep was performed between 0.1% to 100%, with an angular frequency of 1.6 Hz, ω = 10 rad s^−1^, and a 0.3 mm gap between rheometer plates. Oscillatory frequency sweep testing of hydrogels was performed at a constant strain of 0.1% and a frequency ranging from 0.001 to 100 Hz on a logarithmic scale. The temperature sweep measurement was performed in a temperature range from 4 to 40 °C with a ramp of 1 °C min and a frequency of 1 Hz at a constant strain of 0.5%. Finally, the gelation time of the pre-polymer solution was determined with a cone-plate geometry (CP-25, Anton Paar) with a gap of 0.049 mm at 20 °C with a frequency of 1 Hz. Samples are illuminated from the bottom through an X-Cite Series 120 (Excelitas Technologies Corporation, Waltham, MA, USA) system with a wavelength of 365 nm, increasing the shear rate to ≈100 s^−1^ for inducing the gelation. The oscillatory strain sweep has also been evaluated in the cell-laden GelMA-based hydrogels.

##### Rheological Measurements of THA Hydrogels

Oscillatory and rotational (flow curve) tests were performed for THA Hydrogels. For the THA, the gel point, defined as the crossover of the storage modulus G′ over the loss modulus G″, is determined following the G′ and G″ immediately after adding the H_2_O_2_ to the ink precursor. After 30 min, which was needed to ensure the complete cross-linking of the THA hydrogel ink, the viscoelastic linear range was determined by applying a strain sweep range from 0.1% to 100% at a frequency of 1 Hz (amplitude sweep). The damping factor (tan δ) was calculated from the ratio between the loss modulus G″ and the storage modulus G′ values at 1% strain. The cell-laden THA hydrogel was also evaluated by the amplitude sweep.

### 2.5. The 3D Printing of the GelMA-Based and THA Hydrogels

The 3D printing of the developed hydrogel bioinks was carried out using 3D Discovery, Regen HU 3D bioprinter. GelMA hydrogel was prepared and mixed with nHAp at the desired concentration. The hydrogel was filled in a disposable syringe and inserted into an extrusion bioprinter head. Two different needle sizes (0.41 and 0.51 mm ID, Nordson) were used to choose optimal size of the printing needle, the extrusion air pressure ranged between 0.2 and 2.6 psi, and the 3D printing was performed at 20 °C with a deposition speed of 2–10 mm/s. A line with a length of 15 mm was printed on glass slides, which was followed by UV light curing.

THA hydrogel was also prepared at the desired concentration, and the same optimization procedure was performed to optimize different printing parameters at the room temperature; here, the crosslinking was done with the green light.

### 2.6. Characterization of the 3D-Printed Hydrogels

#### 2.6.1. Live/Dead Staining Assay

The viability of hMSCs in the THA and GelMA-based hydrogels was measured with live-dead staining. First, bone marrow aspirates were obtained from patients undergoing spine surgery at the Inselspital Bern (KEK: Req-2016-00141). The Swiss Human Research Act does not apply to research which involves anonymized biological material and/or anonymously collected or anonymized health-related data [30,31]. General Consent which also covers anonymization of health-related data and biological material was obtained from all cell donors. The cells were incubated in α-MEM medium (Sigma) supplemented with 10% FCS (Gibco), 1% of Penicillin/Streptomycin (Gibco), and 0.1% fibroblast growth factor (bFGF) (Peprotech, Rocky Hill, CN, USA). Then, hMSCs at passage three were embedded into the hydrogels with 1 × 10^6^ cells/mL for GelMA-based hydrogels and 5 × 10^6^ cells/mL for THA to prepare the bioinks. After printing a 15 mm long line of each cell-laden bioink into a 24-well plate, the crosslinking was done under UV light for GelMA-based and green light for THA hydrogel, respectively. Afterward, the cell-laden constructs were incubated in α-MEM medium supplemented with 10% FBS. The cell viability was determined by live/dead assay with Calcein-AM/Ethidium homodimer staining at following time points: 2 h, day 1 (24 h), day 3 (72 h), and day 7 (168 h) after printing and cultivation. At a given time point, the culture medium was removed and cell-laden hydrogels were incubated (three constructs per time point and material) in 10 µM Calcein-AM and 2 µM ethidium homodimer solution in PBS for 1 h at 37 °C. After the incubation, hydrogel constructs were analyzed with a confocal laser scanning microscope (LSM510, Zeiss, Jena, Germany), equipped with a digital camera. ImageJ software was used for the measurements of live and dead cells.

#### 2.6.2. Evaluation of Printed Bone and Cartilage Tissue Like Bioink

##### Printing Osteoblasts Embedded in GelMA-nHAp Hydrogel: Bone Part

Primary human cancellous bone osteoblasts (hOBs) were isolated from orthopedic surgery patients according to established protocol [32]. In brief, bone pieces were vigorously washed in PBS and digested with collagenase type IV (Sigma-Aldrich Co.) for 45 min at 37 °C. The digested fragments were placed in 6-well plates and cultivated in low glucose DMEM supplemented with 10% FCS, 1% penicillin-streptomycin, and 50 µg/mL ascorbic acid. The media were refreshed twice a week. Third passage cells were used for the evaluation of the osteoblastic behaviors in GelMA-nHAp bioink.

The hOBs were embedded into GelMA-nHAp hydrogel at a density of 1 × 10^6^ cell/mL. Then, the cell-laden bioink was filled in a sterile disposable syringe and placed into a head of an extrusion bioprinter 3D Discovery (Regen HU, Villaz-Saint-Pierre, Switzerland). After printing a 15 mm long line (with optimized parameters) into a 24 wells plate, the curing was carried out with UV light for 2 min. Further, the printed lines were incubated in an osteogenic medium composed of DMEM 1 g/L glucose supplemented with 10% FCS, 50 µg/mL ascorbic acid 2-phosphate, 5 mM b-glycerophosphate, and 10 nM dexamethasone (all from Sigma-Aldrich), and 100 U/mL of penicillin, 100 μg/mL of streptomycin for maximum 7 days.

##### Printing Chondrocyte-Derived Micropellet Encapsulated in THA Hydrogel: Cartilage Part

First, for the preparation of cartilage mimicking hydrogel bioink, the chondrocyte-derived micropellets were generated by seeding human chondrocytes in a 6-well microwell plate (AggreWell^TM^ 400 plate, Köln, Germany) at a density of 2.1 Mio/well according to the manufacturer’s protocol and cultured for three days in a chondrogenic medium. The chondrogenic medium comprised high-glucose Dulbecco’s Modified Eagle Medium (Gibco) supplemented with ascorbic acid 2-phosphate (50 μg/mL; Sigma-Aldrich), nonessential amino acids (1% *v*/*v*; Gibco), iTS þ premix (1% *v*/*v*; Corning, Arizona, AZ, USA), dexamethasone (100 nM; Sigma-Aldrich), transforming growth factor beta (TGF β1, 10 ng/mL; Fitzgerald, Zurich, Switzerland), and antibiotics (100 U/mL of penicillin, 100 μg/mL of streptomycin; Gibco). Then, the cell micropellets were embedded in THA hydrogel at a final cell concentration of 5 × 10^6^/mL. The THA bioink was also filled in a sterile disposable syringe and placed into an extrusion bioprinter head to print a 15 mm long line in a 24-well plate. Further, the printed cell-laden THA samples were incubated in the above-mentioned chondrogenic medium after curing with the green light for 5 min for more analysis.

##### Live Dead Staining

The viability of hOBs in the GelMA-nHAp and chondrocyte-derived micropellets in the THA hydrogels were measured with Calcein-AM/Ethidium homodimer staining at 2, 24, 72, and 168 h after 3D printing and incubating in a specific medium. The staining procedure was the same as explained in previous sections.

##### RT-PCR Analysis

The expression levels of osteogenic- and chondrogenic-related genes were assessed using quantitative real-time polymerase chain reaction (qRT-PCR) on day 3 and 7 of sample’s incubation in the specific mediums. Total RNA was extracted and purified from the printed GelMA-nHAp and THA hydrogel bioinks using Trizol reagent (Invitrogen, Karlsruhe, Germany) and RNeasy Mini Kit (Qiagen GmbH, Hilden, Germany). Reverse transcription was performed with Superscript VILO cDNA synthesis Kit Manual (Invitrogen Corporation). Relative gene expression qRT-PCR reactions were set up in 10-μL reaction mixtures containing TaqMan Universal Master Mix (Thermo Fisher Scientific, Zürich, Switzerland), the appropriate set of primers, and probes, DEPC-H_2_O, and cDNA template. The expression of IBSP, Coll I, ALP, and Runx-2 were evaluated for the bioink mimicking bone, and Coll II, ACAN, Coll I, Coll X, and Sox9 for the bioink mimicking cartilage and PRLPO was used as a housekeeping gene. The expression of genes was normalized to control encapsulated hOBs into GelMA-nHAp and Micropellet Chondrocyte into THA hydrogels and incubated for 24 h for bone and cartilage bioinks, respectively. Primer and probe sequences are shown in supplementary data Table S1 (Supplementary Materials), while catalog numbers of Assays-on-Demand (Applied Biosystems, Foster City, CA, USA) are listed in the supplementary data Table S2 (Supplementary Material).

##### Histology

The samples were prepared for histological analysis via snap freezing protocols on days 1 and 7. Briefly, samples were fixed in 10% formalin for 24 h before treatment with 30% sucrose in PBS solution for 48 h. After that, the samples were embedded in the tissue freezing component and frozen over liquid nitrogen. Sections were cut on a cryostat (Thermo Scientific, Waltham, MA, USA) with a thickness of 10 μm. Representative sections of osteogenic samples were stained with Van Kossa, and the chondrogenic samples were stained using Toluidine Blue.

### 2.7. Statistical Analysis

Values were reported as mean ± S.D. based on triplicate (*n* = 3). To test the significance of observed differences between the study groups, the two-way ANOVA was applied. A value of *p* < 0.05 was statistically significant. Analyses were carried out using the GraphPad Prism v9.4.0 software (GraphPad Software Inc., La Jolla, CA, USA).

## 3. Results

### 3.1. Characteristics of the Synthesized GelMA: H-NMR

The degree of substitution of the methacryloyl groups in the GelMA samples is identified as the ratio of reacted amino groups to the total amount of original amino groups by H-NMR spectroscopy. The appearance of peaks at 5.5–5.7 ppm in the H-NMR spectrum of GelMA indicates the occurred substitution in the pendant group. H-NMR spectra show an evident decrease in the height of the amino group peaks from lysine at 2.98 ppm when GelMA is compared to gelatin. The peak at 7.2 ppm (aromatic hydrogen) was taken as the reference peak. The area under the peak at 2.8 ppm (lysine amino group) was calculated for both gelatin and GelMA spectra, and DOS of 52.8% was obtained (Figure S1).

### 3.2. Characteristics of the Synthesized THA: H-NMR and UV–Vis Measurement

The THA was successfully synthesized by a coupling reaction between the tyramine amine groups and HA carboxylic acid groups. 1H-NMR and UV–Vis absorbance analysis revealed the successful conjugation of tyramine to HA backbones with a DoF of 6.26 ± 0.02% (Figure S2).

### 3.3. Physicochemical Properties of nHAp

**Figure 1:**
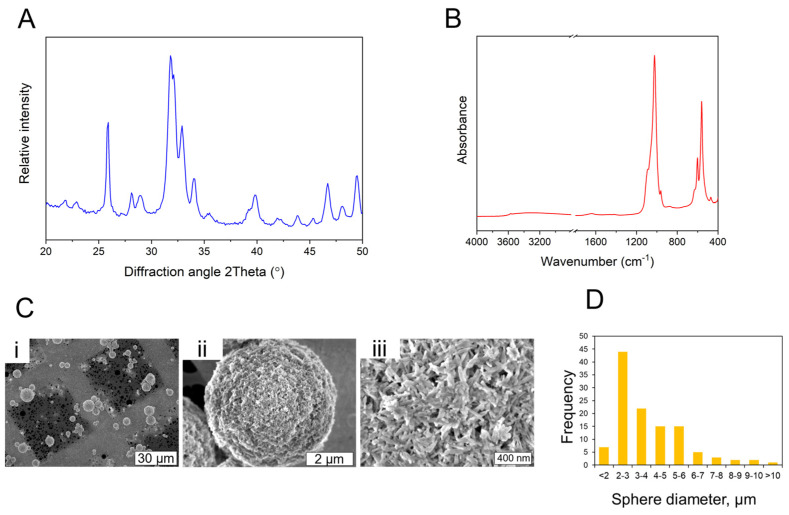
(**A**) X-ray diffraction patterns of spray dried nHAp; (**B**) FT−IR spectrum of the spray-dried nHAp; (**C**) collage of SEM microphotographs (lower and higher magnifications) where an overview of spray-dried nHAp spheres (**i**), a single spray-dried nHAp sphere (**ii**) and morphology of nHAp particles that make up the spray-dried spheres are shown (**iii**); (**D**) size distribution of the spray-dried nHAp spheres.

The chemical element composition of the spray-dried nHAp was determined using ICP-MS (Table S3). The nHAp contains other elements in trace amounts besides calcium and phosphorus, e.g., Mg, Sr, and Zn. The total amount of heavy metals was <9 mg/kg. The XRD pattern of the spray-dried sample (Figure 1A) shows a pattern that is typical to nano-hydroxyapatite with broadened diffraction maxima, indicating the presence of nano-sized particles. The FT-IR spectrum of the spray-dried nHAp is presented in Figure 1B. Overall the spectrum of spray-dried nHAp comprises absorption bands of phosphate groups, carbonate groups hydroxyl group, and adsorbed water. All the above-mentioned chemical groups are characteristic groups of nHAp materials.

The SSA of the spray-dried nHAp was determined to be 83 ± 8 m^2^/g. Particle morphology assessment using both SEM (Figure 1C) and TEM (Figure S3) showed that nHAp has a needle-like particle shape with a length of 81 ± 38 nm and width of 25 ± 4 nm. The average diameter of the spray-dried nHAp spheres was 4 ± 2 μm. The distribution of the spray-dried sphere diameters is shown in Figure 1D. A summary of physicochemical characteristics of the nHAp nanoparticles synthesized using wet chemical precipitation technology is given in Table S4. The spray-dried nHAp was further used to develop and optimize bioink formulations for 3D bioprinting of the bone tissue mimics.

### 3.4. Rheological Properties of GelMA/nHAp Hydrogel Bioinks

Rheological properties play a crucial role in determining printing quality and consistency for the printing process of hydrogel bioinks. Therefore, the following set of rheological measurements was done for GelMA/nHAp hydrogel bioinks: temperature sweep, time sweep, frequency sweep, and amplitude sweep.

According to the temperature sweep measurement, both G′ (storage modulus) and G″ (loss modulus) values decrease upon increased temperature, which confirms the characteristic thermo-responsiveness of GelMA. A single gel–sol transition temperature was determined for GelMA and GelMA/nHAp hydrogels at around 25 ± 0.5 °C. The transition was not affected by the addition of nHAp. For hydrogel bioink to maintain sufficient mechanical strength, self-support, and shape fidelity after 3D printing, the temperature of GelMA/nHAp hydrogel inks should be chosen within a temperature range less than 25 °C in the current case (Figure S4a).

Evaluation of time sweep curves revealed that the values of G′ and G″ were higher in GelMA-nHAp when compared to GelMA; however, the difference was not significant. During the time sweep measurement, the UV light source (λ = 365 nm) was turned on, and thus light cross-linking of the GelMA-based hydrogels was initiated by the photoinitiator Irgacure 2959, which can be observed by an increase in both G′ and G″ values (Figure S4b,c).

Frequency sweep curves are shown in (Figure S4d,e) and the results showed that the storage modulus (G′) at a frequency of 10 Hz was significantly higher for GelMA/nHAp hydrogel than GelMA (significant difference among means detected (*p* < 0.01)).

**Figure 2:**
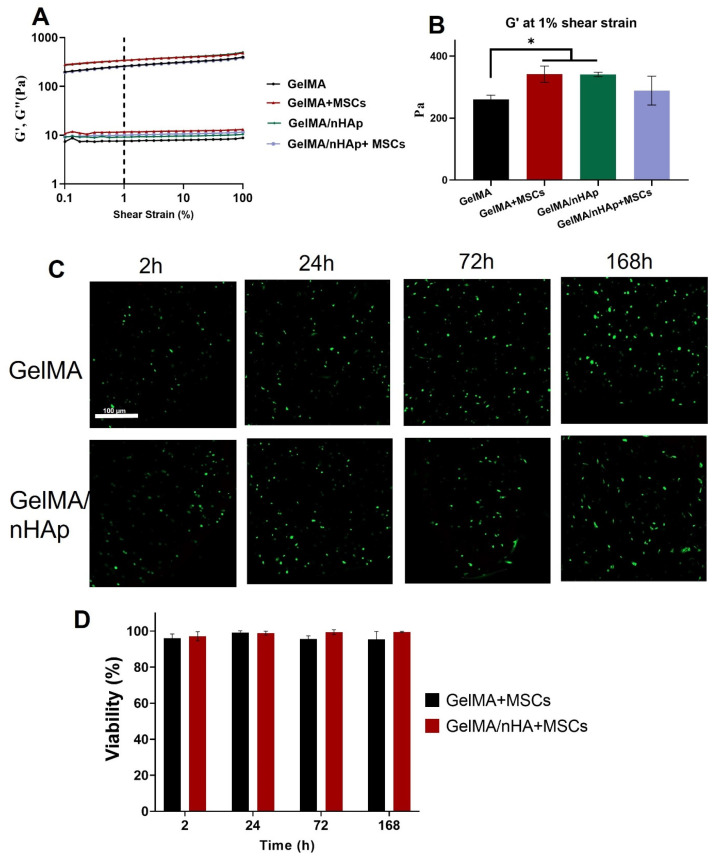
(**A**) Amplitude sweep and (**B**) storage modulus (G′) value extracted at 1% shear strain and visualized in columns for easier identification of differences. * Storage modulus (G′) at 1% shear strain was significantly higher for GelMA/nHAp and cell-laden GelMA hydrogels compared to GelMA hydrogel alone. Here, average of *n* = 3 ± SD is shown; (**C**) viability of GelMA and GelMA/nHAp 1% cell-laden 3D printed constructs with cell density 1 × 10^6^ cells/mL: live-dead staining with Calcein-Am (“green” or live cells) and EthD-1 (“red” or dead cells); (**D**) Cell viability (%) for cellular and acellular printed constructs at different time points.

Amplitude sweep measurements were performed for GelMA-based hydrogels with and without cells (Figure 2A). The results indicated that addition of nHAp into the GelMA hydrogel increases G′ and G″. In addition, storage modulus (G′) at 1% shear strain (Figure 2B) was significantly higher for GelMA/nHAp hydrogels compared to GelMA hydrogel alone. The data also showed the G′ in cell-laden GelMA hydrogel was significantly higher than GelMa without cells, but G′ in the GelMA-nHAp hydrogels did not change by adding the cells (significant difference among means not detected (*p* < 0.05)).

### 3.5. Live/Dead Staining Assay

The cell viability of hMSCs encapsulated into the GelMA-based hydrogels after 3D printing was evaluated at four-time points (2, 24, 72, 168 h) by live/dead staining (Figure 2C). Calcein-AM (green) indicates live cells, while ethidium homodimer-1 (red) indicates the dead cells. The quantified data showed a high percentage of cell viability in both GelMA and GelMA-nHAp hydrogels, and there was no significant difference between the hydrogels (Figure 2D).

### 3.6. THA Bioink Properties

#### 3.6.1. Rheology

**Figure 3:**
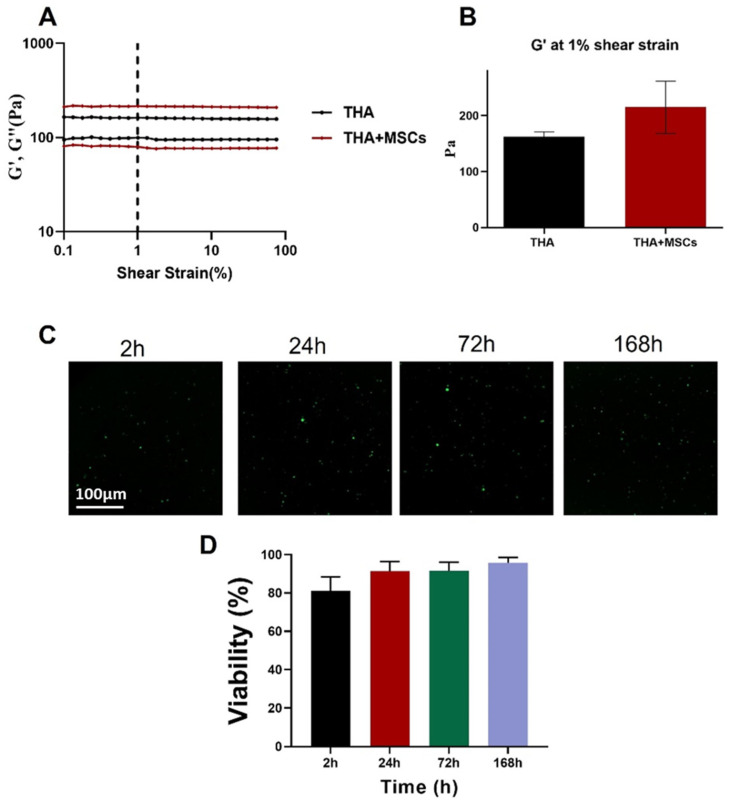
(**A**) Amplitude sweep of THA bioink with 2.5 × 10^6^ hMSCs/mL; (**B**) Value of storage modulus (G′) extracted at 1% shear strain visualized in columns for easier identification of differences. Here average of *n* = 3 ± SD is shown; (**C**) Cell viability of THA 3.5% *w*/*v* cell-laden 3D printed construct with cell density 2.5 × 10^6^ cells/mL live-dead staining with Ca-AM (green, live cells) and EthD-1 (red, dead cells); (**D**) Cell viability (%) for cell-laden THA constructs at different time points.

Comparison of rheologic properties was done on acellular and cellular THA 3.5% *w*/*v* hydrogel bioinks by amplitude sweep measurement, and the G′ value at 1% shear strain was extracted, which both are shown in Figure 3A,B. In Figure 3A the upper curve of each bioink denotes storage modulus (G′), and the lower curve, loss modulus (G″). According to this measurement, no significant difference between G′ in THA hydrogels with and without cells was found.

#### 3.6.2. Live/Dead Staining Assay

The cell viability of hMSCs encapsulated into the THA hydrogel after 3D printing was evaluated at four time points (2, 24, 72, 168 h) by live-dead staining of the cells (Figure 3C). The data showed a high percentage (>95%) of cell viability in the hydrogel after 7 days of cultivation (Figure 3D).

### 3.7. Evaluation of Printed Bone and Cartilage Tissue-Like Bioink

First, all 3D printing parameters such as the needle size, printing speed, printing pressure, and temperature were optimized according to printability and shape fidelity of GelMa-nHAp and THA hydrogel inks in conditions with and without cells. After selecting the optimized parameters, a thin line of GelMA-nHAp encapsulated with osteoblasts was printed for the bone part, and a line of THA encapsulated with chondrocyte micropellets was printed for the cartilage part. The printed bioinks were evaluated for live-dead assay, gene expression, and histology staining.

#### 3.7.1. Live/Dead Staining Assay

**Figure 4:**
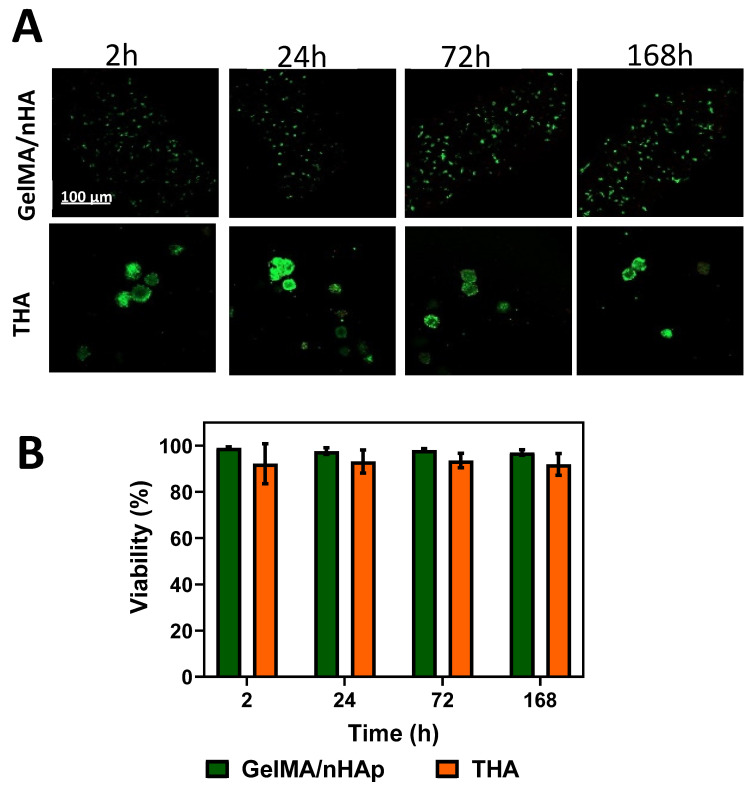
Cell viability of GelMA/nHAp and THA 3D printed constructs: (**A**) live-dead staining with Calcein-AM (“green” or live cells) and EthD-1 (“red” or dead cells) for hOBs embedded in GelMA/nHAp and chondrocyte micropellet encapsulated in THA hydrogel; (**B**) Cell viability (%) for both printed bioinks at four different time points.

The viability of osteoblasts and chondrocyte micropellets into the printed GelMA-nHAp and THA was determined with the live-dead staining at four time points (2, 24, 72, 168 h) after the 3D printing (Figure 4A,B). The results revealed a high percentage of cell viability in both hydrogels at all tested times.

#### 3.7.2. RT-PCR

**Figure 5:**
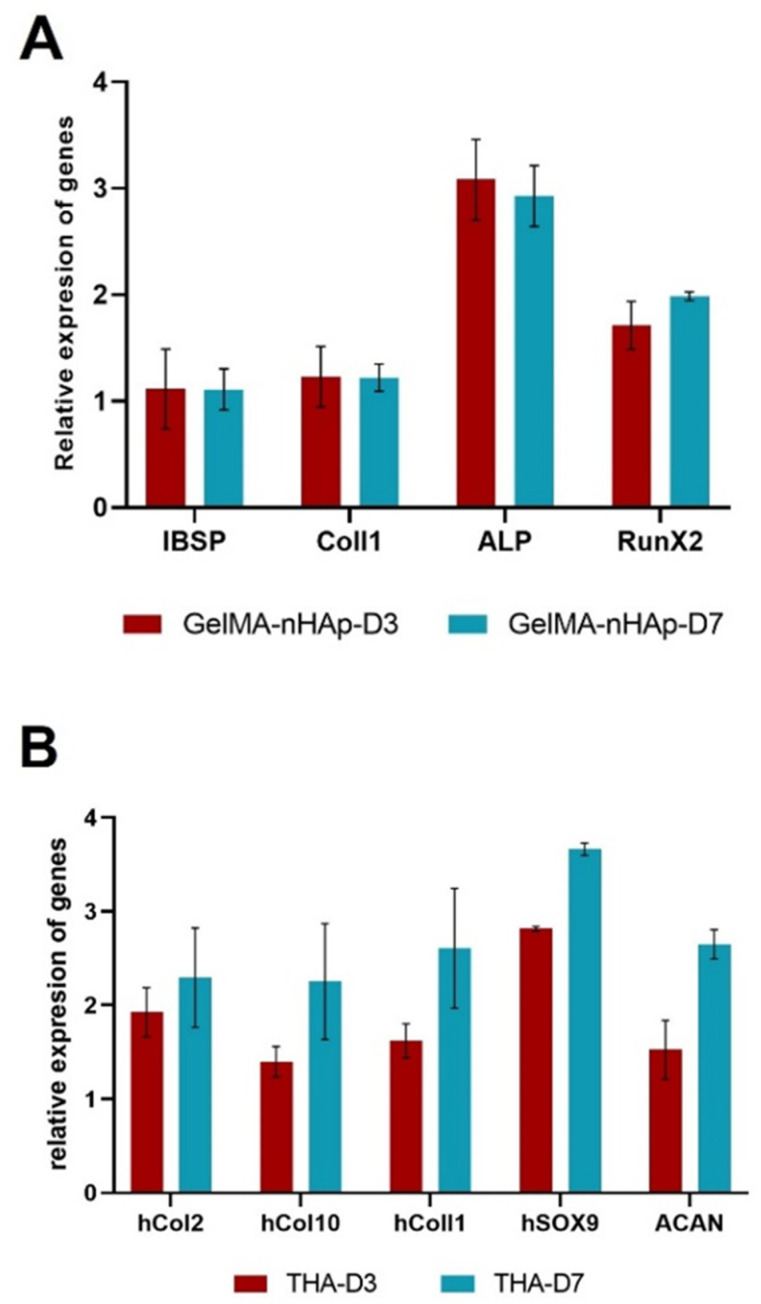
Bone and cartilage-specific gene expression: (**A**) For the 3D printed GelMA/nHAp and (**B**) THA constructs on day 3 and 7 (normalized on day 1).

The real-time PCR was performed to check the maintenance of osteoblast- and chondrocyte-specific gene expression into the printed GelMA-nHAp and THA hydrogels, respectively. The experiment was done with one donor for osteoblast and one donor for chondrocyte cells. The analysis of osteoblast marker genes (Runx2, IBSP, Coll1, ALP) showed constant gene expression on days 3 and 7, and there was no significant difference between them (Figure 5A). The expression of chondrocyte markers (Coll1, Coll2, Coll10, ACAN, and SOX9) showed similar results to bone markers so that the expression of genes on days 3 and 7 was relatively constant (Figure 5B).

#### 3.7.3. Histology

**Figure 6:**
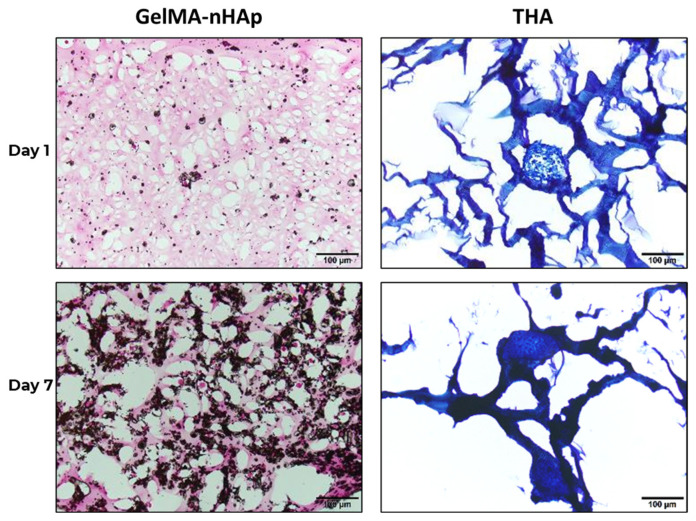
Histological results of GelMA/nHAp and THA 3D printed constructs at day 1 and day 7 after staining with Von Kossa and Toluidine blue stains, scale bar is 100 μm.

Von Kossa staining was used to evaluate the maintenance of osteoblast phenotype during the culture, and the chondrocyte maintenance evaluation was carried out by Toluidine blue staining (Figure 6). Von Kossa staining showed that the GelMA-nHAp progressively darkened to red, indicating the progressive development of mineralization in culture on day 7 compared to day 1. In addition, a darkened blue in THA hydrogels was observed on day 7 compared to day 1 by Toluidine blue staining, which is indicating the improvement of chondrogenesis during the culture.

## 4. Discussion

Development and characterization of new combinations of bioinks for 3D printing is important for the fields of tissue engineering and regenerative medicine, especially for obtaining complex, patient-specific, and multi-layered osteochondral tissue-like cell-laden constructs. In this study, we have successfully developed two hydrogel bioinks made from GelMA-nHAp and THA for extrusion-based 3D bioprinting of bone and cartilage tissue mimics laden with osteoblasts and chondrocytes, respectively.

A previous study revealed that GelMA supports osteogenic differentiation of mesenchymal stem cells (MSCs), confirming its suitability for bone tissue engineering applications [33]. On the other hand, it was demonstrated that hydroxyapatite (Hap) is the most stable of calcium phosphates and chemically and structurally similar to natural apatite in the human bone’s mineral [34]. Furthermore, Hap and nHAp is biocompatible, bioactive, osteoconductive, and osteoinductive [35]. When HAp is added to polymeric composite materials, it usually enhances both in vitro and in vivo behavior of the respective composite material [36]. Therefore, the combination of both GelMA and nHAp leads to a promising composite hydrogel for mimicking the bone tissue in osteochondral defect. Our rheology results of GelMA/nHAp and GelMA hydrogels revealed that the nHAp enforces the viscoelastic properties of GelMA. It has also been reported in previous studies that the addition of HAp to different hydrogels enhanced the mechanical properties and furthermore improved the biological cues [26,34,37].

GelMA, as a widely used biomaterial, has been 3D printed usually at concentrations higher than 10% (*w*/*v*) [24,38]. Due to the thermo-responsive characteristic of GelMA, it does not convert to a printable gel at room temperature in a low concentration, which is also highlighted in the published studies on challenges faced in bioprinting due to the fast sol–gel transition of GelMA bioinks at room temperature [39,40]. Then, we performed the temperature sweep test to find the sol–gel transition point of GelMA-based hydrogel, which was 25 °C and was not affected by adding the mineral component nHAp into the GelMA hydrogel. Therefore, we used the cartridge cooler to set the temperature below the sol–gel transition; in this case, the temperature was set up at 20 °C. GelMA could be printed by adjusting several parameters to find a suitable viscosity like the concentration of the hydrogel, the temperature, and the degree of functionalization [41]. We have found a suitable fabrication window for acellular GelMA/nHAp ink. However, the introduction of living cells into the ink makes the development of such cell-laden 3D printing inks challenging as additional 3D printing parameters and material properties crucial for cell viability need to be considered; that is, the so-called biofabrication window needs to be established [42,43]. However, the effect of encapsulated cells on the viscosity of GelMA is missing. The amplitude sweep test results in the current study showed that by adding MSCs into the GelMA hydrogel, the storage modulus (G′) was increased, which is helpful for 3D printing. It was interesting that the cells did not change the rheological properties in GelMA/nHAp bioink. Perhaps the nHAp already occupied the free space in the GelMA hydrogel, which increased the storage modulus (G′) and by adding the cells, it did not change significantly. In addition, the cell viability in both GelMA and GelMA/nHAp was quite high, verifying the GelMA-based hydrogels’ biological compatibility.

In parallel with GelMA hydrogel development, THA hydrogel was also synthesized and characterized. A dual crosslink process was used to develop the THA bioink as previously introduced by Petta et al. [29]. Initial enzymatic crosslinking by mediated HRP and H_2_O_2_ was used to optimize the extrusion and shape fidelity of the bioink and the second crosslinking with a green light in the presence of EO was introduced to stabilize the shape of the 3D construct.

The rheology assessment showed that by encapsulating MSCs into the THA hydrogel, the storage modules did not change significantly, but it affected printability which was optimized by adjusting the H_2_O_2_ and HRP concentrations. However, the other study has reported that embedding of cells into the THA is correlated with a decrease in storage modulus G′ and printability of the THA bioink. It seems that cells decrease the efficiency of enzymatic cross-linking mediated by HRP/H_2_O_2_, perhaps by scavenging H_2_O_2_ and reducing the di-tyramine bond formation [29].

After developing and characterizing both GelMA/nHAp and THA bioinks, the maintenance of the osteogenic and chondrogenic differentiation was evaluated by encapsulating osteoblasts and chondrocyte micropellets into the GelMA/nHAp and THA hydrogels, respectively. Then, bone and cartilage printed lines were cultivated into the osteogenic and chondrogenic medium for one week. Later, they were investigated by cell viability, specific gene expression, and histological staining.

Live-dead staining for GelMA/nHAp-encapsulated osteoblasts and THA-embedded chondrocyte micropellets again confirmed the biocompatibility of both bioinks. The RT-PCR showed that both GelMA/nHAp and THA hydrogels support and maintain the osteogenic and chondrogenic markers for osteoblasts and chondrocytes, respectively. In addition, the histology staining indicated that both hydrogels could also preserve the osteogenic and chondrogenic differentiation in a short time. As recommended in previous studies, the GelMA-based hydrogel is a well-known hydrogel for osteogenesis and THA for chondrogenesis [33,44].

## 5. Conclusions

In summary, this work developed extrudable bioinks to print the miniature structure with cell-laden GelMA/nHAp and THA hydrogels to mimic bone and cartilage in vitro tissue models. Different parameters such as needle size, printing speed, printing pressure, and printing temperature were optimized for both hydrogels according to extrudability and shape fidelity. It was demonstrated that GelMA/nHAp hydrogels maintain the osteogenic properties of osteoblasts in a short time and in the same way, THA maintained the chondrogenic properties of chondrocyte micropellets, which suggested to develop an in vitro osteochondral model for drug screening to find the best effective drug for osteoarthritis.

## Figures and Tables

**Table 1:** Spray-drying conditions of nHAp slurry.

Diameter of the Nozzle, mm	Inlet Temperature, °C	Outlet Temperature, °C	Feed Flow Rate, mL	Atomization Gas (Air) Flow, L/h
1.5	220	97 ± 5	9	246–357

## Data Availability

All data can be requested from the corresponding authors.

## Supplementary Materials

The following supporting information can be downloaded at: https://www.mdpi.com/article/10.3390/ma16227214/s1: Figure S1: H-NMR spectra of (a) Gelatin and (b) GelMA; Figure S2: H-NMR spectrum of THA; Figure S3: TEM images of spray-dried nHA; Figure S4: Rheological Properties of the GelMA based hydrogel; Table S1: Primer and probe sequences for qRT-PCR; Table S2: List of assays on demand used for qRT-PCR; Table S3: The concentration of chemical elements in nHA besides Ca and P; Table S4: Characteristics of nHA synthesized by wet precipitation technology.
