# Supplementary material for: Cell-Laden 3D Printed GelMA/HAp and THA Hydrogel Bioinks: Development of Osteochondral Tissue-like Bioinks
Source: Materials (Basel). 2023 Nov 17;16(22):7214. doi: 10.3390/ma16227214 (PMC10673417; doi:10.3390/ma16227214)
Supplement: Supplementary file 1 [file materials-16-07214-s001.zip › materials-2652884-supplementary.pdf]

Supporting Information

Cell-Laden 3D Printed GelMA/HAp and THA Hydrogel Bioinks: Development of Osteochondral Tissue Like

Table S1. Primer and probe sequences for qRT- PCR.

Gene	Forward primer	Reverse primer	Probe
Coll 2	5' – GGC AAT AGC AGG TTC ACG TAC A -3'	5'- GAT AAC AGT CTT GCC CCA CTT ACC -3'	5'- CCT GAA GGA TGG CTG CAC GAA ACA TAC -3'
Coll 10	5'- ACG CTG AAC GAT ACC AAA TG -3'	5'- TGC TAT ACC TTT ACT CTT TAT GGT GTA -3'	5'- ACT ACC CAA CAC CAA GAC ACA GTT CTT CAT TCC -3'
Coll 1	5'- CCC TGG AAA GAA TGG AGA TGA T -3'	5'- ACT GAA ACC TCT GTG TCC CTT CA -3'	5'- CGG GCA ATC CTC GAG CAC CCT -3'
ACAN	5'- AGT CCT CAA GCC TCC TGT ACT CA -3'	5'- CGG GAA GTG GCG GTA ACA -3'	5'- CCG GAA TGG AAA CGT GAA TCA GAA TCA ACT -3'
Sox 9	5'- TCT GGA GAC TTC TGA ACG AGA G -3'	5'- CTG GTA CTT GTA ATC CGG GTG -3'	5'- TCC ACG AAG GGC CGC TTC T -3'
IBSP	5'- GGC CTG TGC TTT CTC AAT G -3'	5'- CGT GGC CTG TAC TTA AAG ACC CCA TT-3'	5'- ACT GCC CTG AAC TGG AAA TC-3'
Runx2	5'- AGC AAG GTT CAA CGA TCT GAG AT -3'	5'- TTT GTG AAG ACG CTT ATG GTC AA -3'	5'-TGA AAC TCT TGC CTC GTC CAC TCC G-3'
RPLP0	5'- TGG GCA AGA ACA CCA TGA TG-3'	5'- CGG ATA TGA GGC AGC AGT TTC-3'	5'- AGG GCA CCT GGA AAA CAA CCC AGC -3'

Table S2. List of assays on demand used for qRT-PCR.

Gene	Assay ID
Human ALPL	Hs01029144_m1

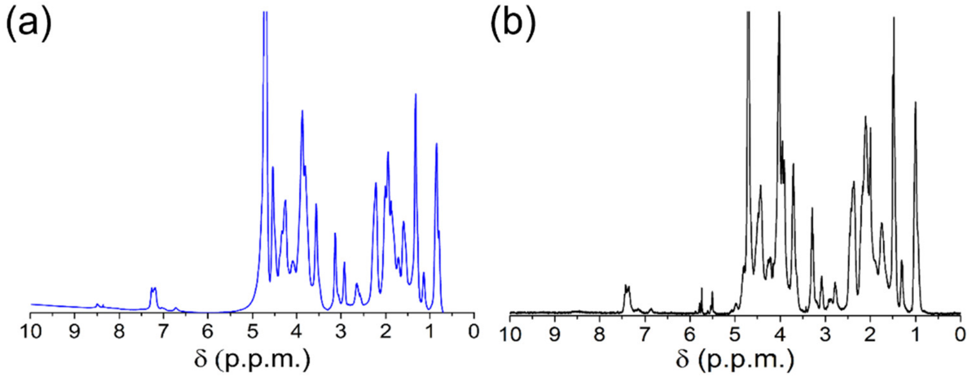

Figure S1. H-NMR spectra of a) Gelatin and b) GelMA.

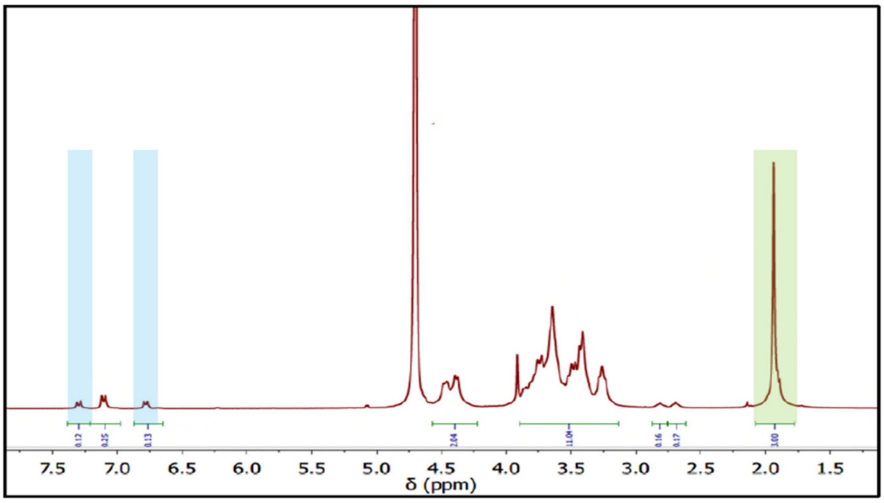

Figure S2. H-NMR spectrum of THA. The peak around 1.9 ppm, marked in green, represents 3 protons, and the peaks around 6.8 and 7.3 ppm are marked in blue. Each represents two protons caused by tyramine functionalization of HA (280 kDa).

Table S3. The concentration of chemical elements in nHA besides Ca and P.

Element	Concentration
Mg	0.038 ± 0.004 g/kg
Sr	124 ± 12 mg/kg
Zn	3.55 ± 0.36 mg/kg
K	< 0.02 g/kg
Na	< 0.02 g/kg

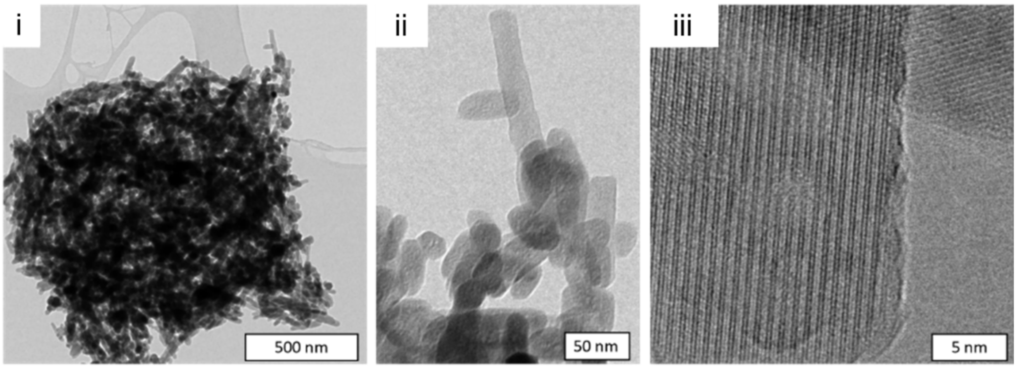

Figure S3. TEM images of spray-dried nHA: (i) view of one spray-dried nHA sphere, (ii) close-up of nHA rounded particles, (iii) high-resolution TEM image of nHA crystallographic planes.

Table S4. Characteristics of nHA synthesized by wet precipitation technology.

Ca/P molar ratio	1.67 (stoichiometric HAp)
Specific surface area	83 ± 8 m^2/g
Particle shape	Needle like
Length x width of nHA particles	81 ± 38 x 25 ± 4 nm
Size of the spray-dried nHA spheres	4 ± 2 µm

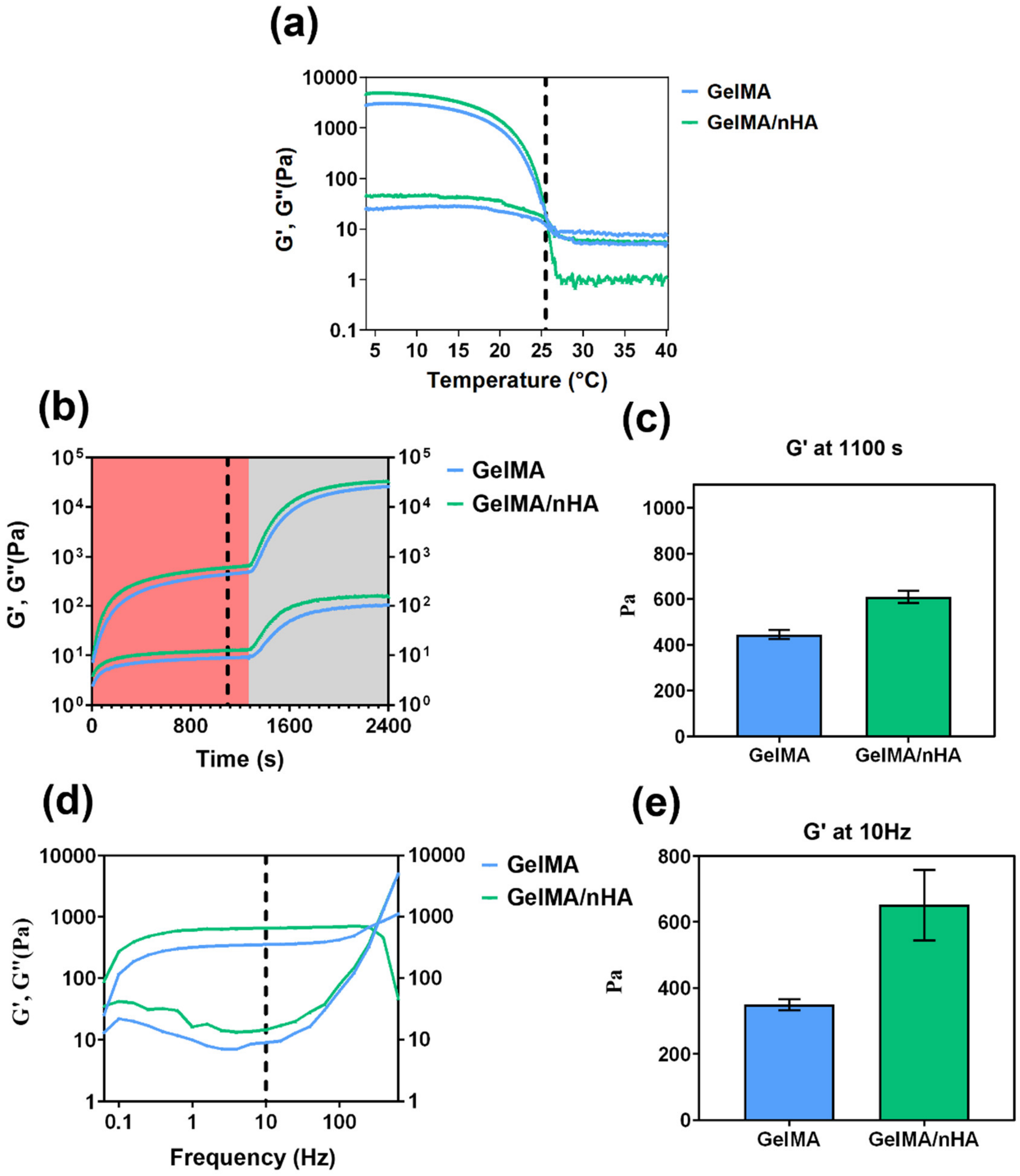

Figure S4. Rheological Properties of the GelMA-based hydrogel. a) Variation in dynamic storage modulus G′ and loss modulus G″ as a function of temperature in a temperature sweep test of GelMA and GelMA/nHA 1% hydrogels, where the cross-over points between the gel and sol state (G′= G″) represent the gel-sol transition temperature marked by a vertical dashed line at 25 °C. All measurements were done in triplicate (n=3); b) time sweep measurements, where the red shaded region indicates was taken at 20°C, and the grey shaded region indicates the time when UV light was turned on to allow effective crosslinking of GelMA in the presence of Irgacure 2595 and c) storage modulus (G') value extracted at a time point of 1100 s. Here average of n=3 ± SD is shown; d) frequency sweep and e) storage modulus (G') value extracted at 10 Hz visualized in columns for easier identification of differences. Here average of n=3 ± SD is shown.
